# Post-Operative Radiation in Early Breast Cancer with N1 Disease: 10-Year Follow-Up

**DOI:** 10.3390/diseases12070145

**Published:** 2024-07-05

**Authors:** Ee Ling Serene Tang, E-Jan Sim, Wei-Wen Ang, Jun Su, Juliana Jia Chuan Chen, Mun Yew Patrick Chan, Bok Ai Choo, Ern Yu Tan

**Affiliations:** 1 Department of Surgery, Woodlands Health, Singapore 737628, Singapore; 2 Department of General Surgery, Tan Tock Seng Hospital, Singapore 308433, Singaporeern_yu_tan@ttsh.com.sg (E.Y.T.); 3 Department of Radiation Oncology, Icon Cancer Centre, Singapore 574623, Singapore; 4 Lee Kong Chian School of Medicine, Nanyang Technological University, Singapore 639798, Singapore; 5 Institute of Molecular and Cell Biology, Agency for Science, Technology and Research (A*STAR), 61 Biopolis Street, Singapore 138673, Singapore

**Keywords:** nodal radiation, recurrence, survival

## Abstract

**Simple Summary:**

Post-operative radiotherapy for post-menopausal women with early breast cancer and small-volume axillary lymph node involvement (N1 disease) is still controversial. The aim of our retrospective study is to assess the 10-year overall survival and disease-free survival of patients who received radiotherapy versus those who did not receive radiotherapy. We also aimed to evaluate factors that affected the use of radiotherapy. Our results revealed no significant differences in the 10-year overall survival and disease-free survival. There was, however, a significant improvement in 10-year overall survival in patients who had radiotherapy if hormonal therapy was received. Radiotherapy appears to be beneficial in improving overall survival in post-menopausal patients with N1 disease and early breast cancer, who are on hormonal therapy.

**Abstract:**

Post-operative radiotherapy for post-menopausal women with early breast cancer and N1 disease is controversial. Although locoregional control is improved, overall survival (OS) benefit is unclear. The clinical benefit of post-operative irradiation in this group of patients over 10 years was reviewed. We aimed to evaluate the OS, disease-free survival (DFS), and factors affecting OS and DFS. A retrospective review of 191 post-menopausal women with early breast cancer and N1 disease from 2004 to 2011 was performed. Demographics, post-operative histology, adjuvant treatment, OS, and DFS were evaluated. Post-operative radiation was given to 95 of 191 women (49.7%). Younger age at diagnosis (*p* < 0.001), a greater number of involved nodes (*p* = 0.004), lymphovascular invasion (LVI), and a higher tumor grade (*p* = 0.001) were more likely in women who received post-operative radiation. Nodal radiation did not improve 10-year DFS (*p* = 0.084) or OS (*p* = 0.203). Post-operative nodal radiation was associated with significant improvement in 10-year OS in women who received only hormonal therapy (*p* = 0.047) and no other systemic therapy. Women with unfavorable risk factors were more likely to receive post-operative radiation, likely due to a perceived higher risk of recurrence. Nodal radiation did not significantly improve 10-year DFS or OS in early breast cancer patients with N1 disease, and the benefit was not clearly demonstrated. However, in those who were on hormonal therapy, radiotherapy was beneficial in improving overall survival.

## 1. Introduction

More aggressive multimodality treatments have improved survival outcomes in women with breast cancer, but these have also increased treatment-related complications as well as the financial and psychosocial burdens of treatment. As the indications for treatments become more inclusive, greater emphasis is being placed on more precise estimations of the benefit-risk ratio. More women are now being recommended adjuvant radiation. Radiation duration has decreased over the years, improving patients’ treatment burden [1]. Radiation to the breast has long been established as an essential modality after breast-conserving surgery and when the residual disease is anticipated, as in the case of margin involvement, large locally advanced tumors, and chest wall involvement. Nodal radiation follows the same principles and has been recommended when there is extensive nodal involvement. Nodal radiation reduces the risk of locoregional recurrence beyond that achievable with systemic treatment alone, thereby suggesting that radiation is necessary to eliminate tumor foci remaining after axillary dissection [1,2]. Although it is debatable whether systemic relapse is a direct consequence of untreated locoregional recurrence, the two are strongly correlated, and optimal locoregional control improves overall survival [2,3,4]. Apart from improving locoregional control, nodal radiation appears to also have an effect on distant disease control [5,6]. Post-operative nodal radiation has been increasingly recommended to women with N1 disease (one to three involved nodes) following data from the EBCTCG meta-analyses that demonstrated a clear benefit regardless of the extent of nodal involvement [2]. The Cochrane database reported an improvement in locoregional recurrence and overall survival, although most of the studies were conducted in the 1980s, and only one study pertained to modern-day radiotherapy practice [7]. However, more recent studies have found that the reductions in locoregional and distant events have not produced significant improvements in overall survival, and questions are being raised about the justification for radiation in women with low-burden N1 disease [5,6,8]. More recently, in 2023, Kim et al. reported no significant differences in 8-year overall survival and locoregional recurrence and suggested the omission of radiation in T1-2N1 breast cancer patients [9]. Definitive recommendations for post-operative nodal radiation in post-menopausal women with N1 disease (one to three nodes involved) are still awaiting the SUPREMO trial.

The 2022 NCCN guidelines recommend regional nodal irradiation to patients with N1 disease, with whole breast or chest wall irradiation, depending on whether breast-conserving surgery or mastectomy was performed [10]. ESMO guidelines on early breast cancer did not clearly recommend but asked for post-mastectomy radiotherapy to be considered in patients with N1 disease [11]. There are, however, other studies that recommend post-operative nodal radiation only for N2 disease (four or more involved nodes), and also for N1 disease in pre-menopausal women [2,12]. Recurrence risk is positively correlated with age and is, by comparison, lower in older post-menopausal women. Consequently, the benefit of post-operative nodal radiation after full axillary nodal dissection is still considered borderline in post-menopausal women with N1 disease [13,14,15,16]. Concerns are that the additional benefit of radiation may be too small to justify the potential morbidity, such as dermatitis, lymphedema, pneumonitis, cardiac injury, and late secondary cancers [5,17]. This has particular relevance to our local practice as many women eligible for breast conservation, in particular older post-menopausal women, opt for mastectomy in order to avoid post-operative radiation [18]. Expanding the recommendations for post-operative nodal radiation may, therefore, influence the surgical decision-making process as the perceived advantage of mastectomy will now be smaller given the higher probability of requiring post-operative radiation. The burden of treatment is another important consideration as regimens in use at our unit require daily sessions for up to 4 weeks. Many older women lack the confidence to attend these sessions alone and rely on a caregiver, thereby further adding to the economic burden of treatment.

In this study, we have evaluated the clinical outcomes of post-operative nodal radiation in post-menopausal women with N1 disease and early breast cancer. Primary endpoints include disease recurrence and overall survival. We sought to identify factors that were more common among women treated with post-operative nodal radiation in order to better understand the practice among our radiation oncologists and also sought to identify a subgroup of women who would derive a greater magnitude of benefit from post-operative nodal radiation.

## 2. Materials and Methods

A retrospective review was performed of 191 post-menopausal women who were staged as having N1 disease (1 to 3 nodes involved) following curative breast cancer surgery at our institute from 1 January 2004 to 31 December 2011. This study was granted approval by the institutional ethics board (DSRB 2014/00546). All women who had undergone curative surgery, either wide local excision (WLE) or mastectomy, with or without breast reconstruction, had N1 disease, and had stage 1 or 2 breast cancer were included. Male patients, those who had received neoadjuvant treatment, those who presented with metastatic disease, and those with ductal carcinoma in situ (DCIS) were excluded.

Full axillary lymph node dissection (ALND), including the level I and II nodes, was performed in all instances. If there was no clinical or radiological evidence of nodal involvement, a sentinel lymph node (SLN) biopsy was first performed, followed by ALND when the SLN was positive for metastases. This was generally performed in the same setting as it is our practice to evaluate the SLN with an intra-operative frozen section [19]. In cases of a false negative frozen section analysis, repeat surgery for ALND was discussed on a case-on-case basis. If there was pre-operative histological confirmation of nodal involvement, ALND was performed upfront without SLNB. Level III nodes were not routinely included in the ALND unless the level III nodes were clinically palpable during surgery or when there was radiological evidence of level III nodal involvement.

Post-operative whole breast radiation was recommended for all women who underwent WLE. Whole breast irradiation comprising 2-field with a total of 50 Gy was administered in 25 fractions; an additional boost of 10 Gy was given to the tumor bed and a higher boost of 16 Gy was given in instances where the margins were inadequate or where there was skeletal muscle involvement. For those who had undergone a mastectomy (with or without reconstruction), chest wall irradiation of 50 Gy in 25 fractions was given, with no additional boost. All patients were simulated supine with both arms abducted above 90 degrees towards the head and with both hands holding the handles of the armrest. The head was turned away from the side of treatment. Computed tomography slices were obtained at 5 mm intervals from the temporomandibular joint to the mid umbilicus, and radiopaque markers were placed on the chest wall central mastectomy scar and 2 cm above and below the previously resected breast margins using the contralateral breast as a guide. Further markers were placed in the midline at the sternum and mid-axillary line. For nodal radiation, the level I, II, and III (supraclavicular) nodes were included (3-field). An additional field to cover the posterior axilla (4-field) was considered in cases of extranodal extension. The internal mammary nodes (IMNs) were not routinely included in the radiation fields unless for medial tumors or when there was prior radiological evidence of IMN involvement. A conventional 3-field plan using a mono isocentric technique with 2 tangential fields and a matching single direct anterior supraclavicular field angled 10 degrees off the spinal cord was used to treat the ipsilateral chest wall and lymph nodes. The total dose prescribed was 50 Gray in 25 fractions with 6 MV photons. The borders of the chest wall were defined medially as midline, laterally at the mid axillary line, superiorly at the suprasternal notch, and inferiorly, at 2 cm below the previous resected breast using the contralateral breast as a guide. The borders of the supraclavicular field were medially at the midline, the lateral border at the lateral coracoid process, superiorly at the inferior aspect of the thyroid cartilage, and inferiorly at the matched superior junction of the tangential beams. Adjuvant treatments were discussed at the multidisciplinary tumor board meetings, where the NCCN guidelines were adopted. These patients would be routinely recommended chemotherapy (anthracycline- and taxane-based regimens) unless there were medical contraindications. Trastuzumab was recommended for HER2-positive tumors and hormonal therapy for hormone-responsive tumors.

Data were collected from patients’ clinical records and demographic data, clinical presentation, type of surgery performed, histological analyses, treatment received, and outcome parameters were included. Details of the radiation treatment regimens were also recorded. Correlation analyses were performed using the Chi-squared test and Fisher’s test where appropriate. Comparison between groups was performed with the Mann–Whitney test and one-way ANOVA as appropriate. Univariate analyses were performed with GraphPad Prism version 10 (GraphPad software Inc., San Diego, CA, USA). Logistic regression was used to identify independent factors associated with post-operative nodal radiation, recurrence-free survival, and overall survival, and was carried out using the Stata package release 11.0 (Stata Corporation, 4905 Lakeway Drive, College Station, TX, USA). Correlation with recurrence-free and overall survival was analyzed with Kaplan–Meier survival curves using Graphpad Prism version 10 (GraphPad software Inc., San Diego, CA, USA). A 2-tailed *p* value test was used and a *p* value of less than 0.05 was considered statistically significant.

## 3. Results

In this study, we reviewed 191 women with stage I or II invasive breast cancer and N1 involvement (one to three positive nodes). All women were post-menopausal and the median patient age was 61 years (ranging from 50 to 90 years). The majority of the women were Chinese (84.3%). Most tumors were classified as invasive ductal carcinoma or not otherwise specified (179 of 191, 93.72%), seven tumors (3.70%) were classified as invasive lobular carcinoma, two (1.05%) as mucinous carcinoma, one (0.52%) as papillary carcinoma, one (0.52%) as metaplastic carcinoma, and one (0.52%) as invasive neuroendocrine carcinoma. The median tumor size was 24 mm (ranging from 2 to 50 mm) and the median tumor grade was grade 3. Seventy-six percent (146 of 191) of patients had ER-positive tumors and 25.4% (35 of 138) of patients had HER2-positive tumors (HER2 status was unknown in 53 patients as HER2 testing became routine only in 2006). More than half the women (135 of 191, 70.7%) had undergone a mastectomy, and all women underwent full axillary nodal dissection, as was the practice at our unit during the study period. A median of 20 nodes (ranging from 5 to 50) was harvested and there were only 13 instances where less than 10 nodes were harvested. A single positive axillary lymph node was found involved in 112 of 191 (58.6%) women.

Fifty percent (95 of 191) of women received post-operative nodal radiation therapy, half of whom (47 of 95) had been treated with WLE. Only 9 of the 56 patients treated with WLE declined radiation therapy. Young age at presentation (*p* < 0.001), WLE (*p* < 0.001), unfavorable tumor features, including a greater number of involved nodes (*p* = 0.004), higher tumor grade (*p* = 0.001), lymphovascular invasion (LVI) (*p* = 0.001), and estrogen receptor-negative status (*p* = 0.025) were positively associated with post-operative nodal radiation (Table 1).

**Figure 1:**
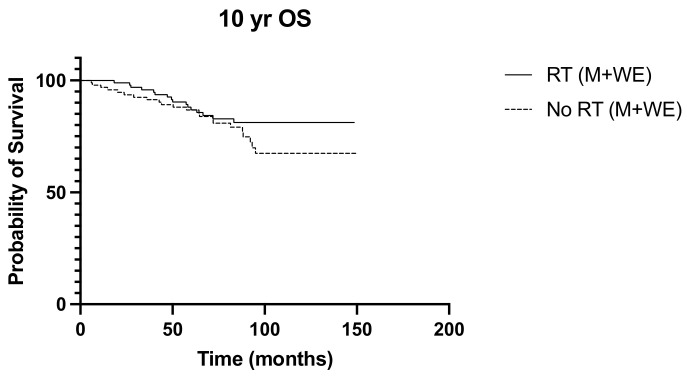
10-year OS RT vs. No RT (M + WLE), *p* = 0.203, HR 0.660, 95% CI 0.349 to 1.25.

**Figure 2:**
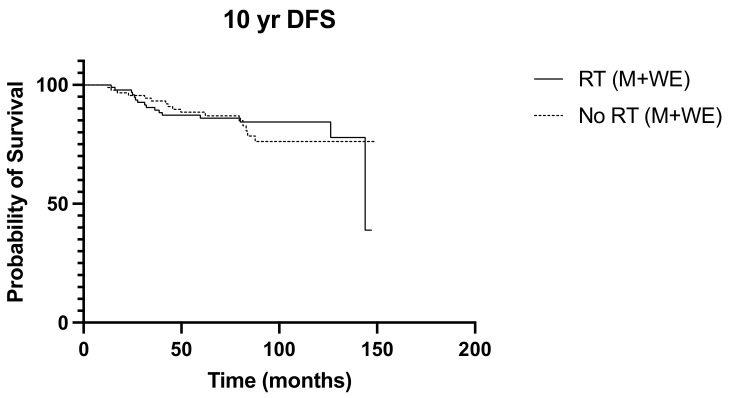
10-year DFS RT vs. No RT (M + WLE), *p* = 0.084, HR 0.902, 95% CI 0.450 to 1.81.

**Figure 3:**
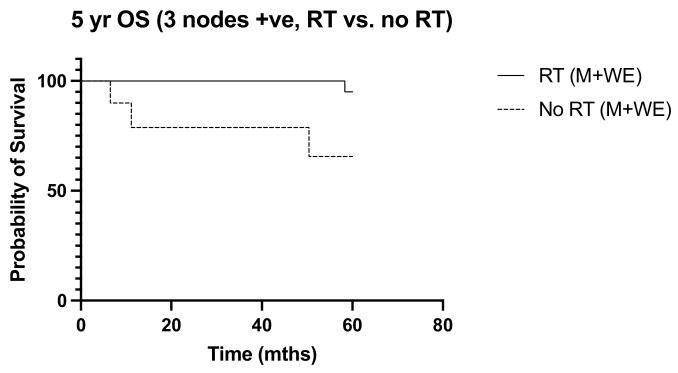
5-year OS, 3 nodes +ve, RT vs. No RT (M + WLE), (n = 33)*, p*=0.015, HR 0.057, 95% CI 0.006 to 0.577.

**Figure 4:**
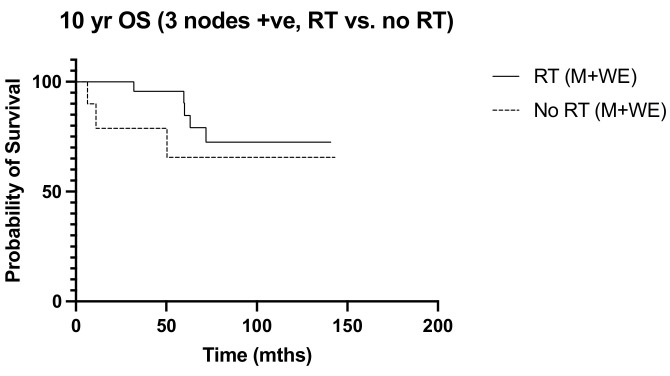
10-year OS. 3 nodes +ve: RT vs. No RT (M + WLE), (n = 33) *p* = 0.321, HR 0.432, 95% CI 0.082 to 2.27.

Over a median follow-up period of 155 months (ranging from 5 to 228 months), locoregional recurrence developed in 32 patients (16.7%), and there were 38 deaths (19.9%). The frequency of recurrence and deaths in the 10 years from diagnosis were similar between the irradiated and non-irradiated groups (*p* > 0.999 and *p* = 0.365, respectively). Radiation was not associated with significant survival benefits in N1 disease, with the 10-year overall survival and disease-free survival curves of the irradiated and non-radiated groups largely overlapping (*p* = 0.203 and *p* = 0.084, respectively) (Figure 1 and Figure 2). The 5-year OS was noted to be significantly improved in those with three positive nodes, who had radiation, compared to no radiation (*p* = 0.015) (Figure 3). However, this improvement in overall survival tailed off and was not observed at 10 years, when there was no difference in the outcome of both groups (*p* = 0.321) (Figure 4). There was no difference in the disease-free survival in both groups at 5 and 10 years.

### 3.1. Factors Affecting Decision for Nodal Radiotherapy

**Figure 5:**
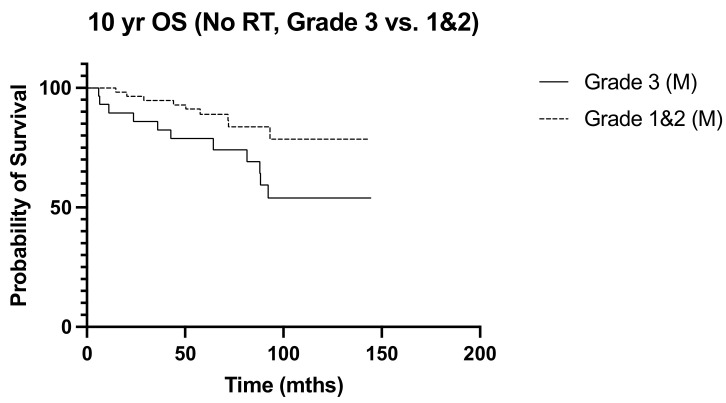
No RT: 10-year OS Grade 3 vs. Grades 1 and 2 (M). (n = 134), *p* = 0.040, HR 2.66, 95% CI 1.05 to 6.76.

**Figure 6:**
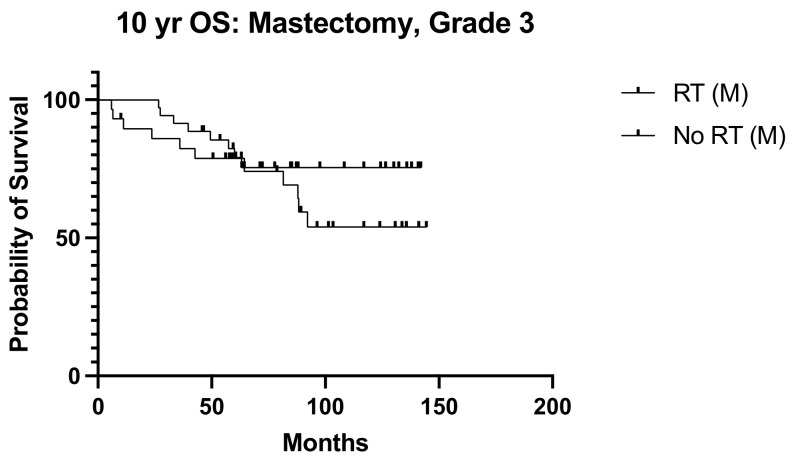
10-year OS Grade 3, M only, RT vs. no RT, (n = 135) *p* = 0.251, HR 0.587, 95% CI 0.237 to 1.46.

**Figure 7:**
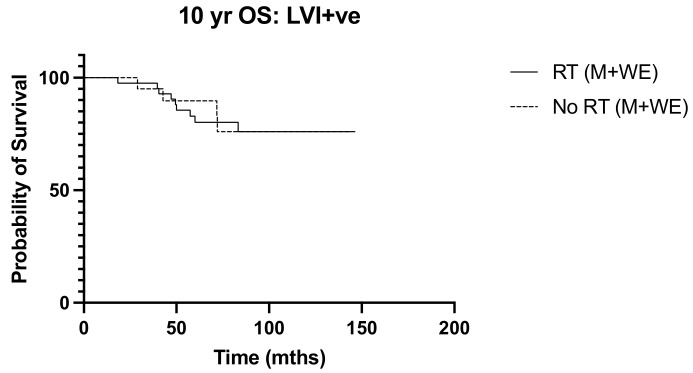
10-year OS in lymphovascular invasion (LVI) positive tumors, RT vs. No RT (n = 63). *p* = 0.909, HR 1.07, 95% CI 0.334 to 3.43.

**Figure 8:**
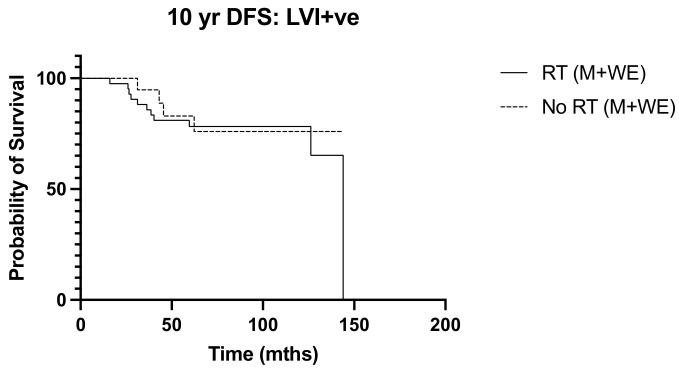
10-year DFS in lymphovascular invasion (LVI) positive tumors, RT vs. No RT (n = 63). *p* = 0.845, HR 1.12, 95% CI 0.359 to 3.50.

We next sought to identify women with N1 disease at high risk of recurrence who would be expected to derive a greater benefit from post-operative nodal radiation. High tumor grade (*p* = 0.013, OR 3.038, 95% CI 1.261–7.317) and lymphovascular invasion (*p* = 0.017 OR 2.975, 95% CI 1.215–7.286) emerged as independent predictors of recurrence (Table 2). Ten-year overall survival was worse among women with high tumor grade undergoing mastectomy (*p* = 0.040, HR 2.660, 95% CI 1.05–6.76) (Figure 5), but the addition of post-operative nodal radiation did not improve overall survival (*p* = 0.251) (Figure 6). Subgroup analysis of patients with lymphovascular invasion did not reveal any worse 10-year OS (*p* = 0.909) or DFS (0 = 0.845) outcome (Figure 7 and Figure 8). No significant association of poorer outcome was observed with the number of involved nodes (*p* = 0.321) and tumor ER negativity (*p* = 0.895), factors that were often taken into consideration during the discussion for post-operative nodal radiation (Table 3).

### 3.2. Effect of Nodal Irradiation in Patients Undergoing Systemic Therapy

**Figure 9:**
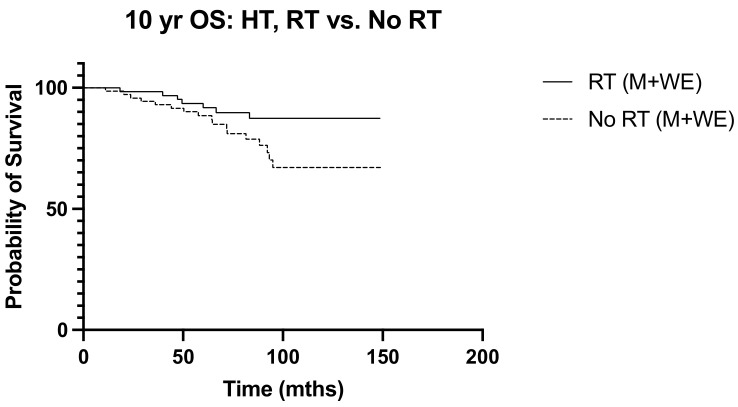
10-year OS in HT gp: RT vs. No RT (n = 134) (M + WLE), *p* = 0.047, HR 0.443, 95% CI 0.199 to 0.988.

**Figure 10:**
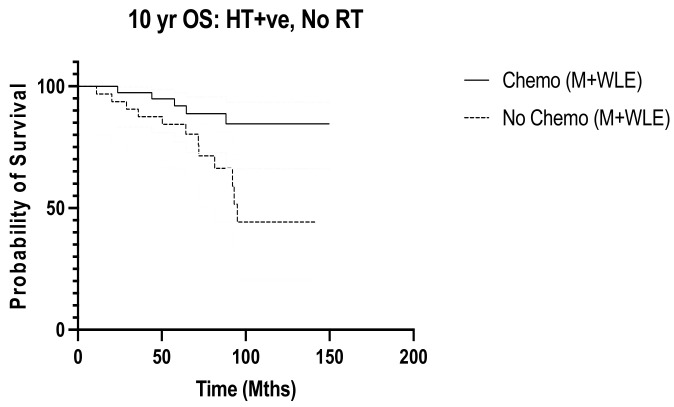
10-year OS in hormonal therapy group with no RT, Chemo vs. no Chemo (n = 71), *p* = 0.010, HR 0.279, 95% CI 0.105 to 0.739.

Next, we determined the benefit of post-operative nodal radiation in relation to the other treatments that would have been recommended to most women with N1 disease. The addition of radiation to women receiving hormonal therapy was associated with significant improvement in 10-year overall survival (*p* = 0.047) (Figure 9). There was no effect of radiation on 10-year disease-free recurrence in this group of women (*p* = 0.493). No additional benefit from radiation was seen in those receiving chemotherapy or targeted therapy. Of the 134 women who received hormonal therapy, 93 (69.4%) also underwent chemotherapy. In women who had hormonal therapy but no radiation, 10-year overall survival was poorer in those who had no chemotherapy (*p* = 0.010, HR 0.279, 95% CI 0.105 to 0.739) (Figure 10). When we further evaluated those who had no chemotherapy but had hormonal therapy alone (n = 41), radiotherapy trended towards improved 10-year overall survival, although this was not statistically significant (*p* = 0.072). There was no effect on disease-free survival (*p* = 0.237).

In total, 18 women did not receive any form of systemic therapy; 3 of these 18 women received post-operative radiation, and none developed distant recurrence. In addition, 15 women had no treatment apart from surgery, with 2 of the 15 women relapsing with systemic disease (13.3%). These two women only had a single involved node each; tumor size was 22 mm in one and 38 mm in the other. One tumor was triple negative and the other was ER positive, PR negative, with unknown HER2 status.

## 4. Discussion

For women treated with breast conservation, post-operative nodal irradiation does not aggravate potential toxicities nor change the dosing schedules significantly. This is not the case for those who have had mastectomy, and has important implications in our local practice where many women eligible for breast conservation opt instead for mastectomy in order to avoid post-operative radiation [18]. A definite survival benefit is conferred by radiation in N2 and N3 disease, and nodal radiation is part of standard guideline recommendations [12], but the benefit is less definite in N1 disease. A reduction in locoregional recurrence has been observed in the literature. However, while nodal radiation also seemed to control systemic recurrence, an overall survival advantage has not been consistently demonstrated [2,5,6,8,20,21]. Our study did not reveal any significant 10-year overall survival or any disease-free survival benefit in post-menopausal women who underwent radiation.

Pre-menopausal women with N1 disease, deemed to have higher recurrence risks, are routinely recommended post-mastectomy radiation, but the benefit is less clear in older post-menopausal women. Unfortunately, studies have not specifically evaluated the benefit in this group of women; those evaluating nodal radiation in N1 disease included both post- and pre-menopausal women, and 9% of the women in the Danish DBCG 82b and 82c studies, evaluating post-mastectomy radiation in post-menopausal women, had high-risk node-negative disease [20,21,22,23]. A further issue raised with regard to these older studies is that the reported locoregional recurrence rates were much higher compared to current standards [24,25,26,27]. Axillary dissection was less extensive in these earlier studies and the treatment regimens used then are no longer considered first-line. Anthracyclines and taxanes were seldom used, and neither aromatase inhibitors nor trastuzumab were available then. Even the dosing schedule of tamoxifen was different; while tamoxifen is now recommended for 10 years, a 5-year regimen was the recommendation then, and in the Danish DBCG 82c study, tamoxifen was given as a 30 mg daily dose for 1 year [22]. Systemic treatment has since improved significantly. Comparison between studies is also confounded by institutional variability in the nodal radiation field coverage and techniques. Radiation fields included the internal mammary nodes (IMNs) in several studies [5,6], which are not our standard practice, and the IMNs are only included for medial tumors or when there is evidence of IMN involvement. Routine inclusion of the IMN remains controversial. Isolated IMN metastases and failure are very uncommon [6,17,28], although the recent DBCG-IMN study demonstrated an overall survival advantage and disputed the long-held concern of increased cardiotoxicity [29].

In the Danish DBCG 82c study, evaluating postmenopausal women with tamoxifen alone versus tamoxifen with radiotherapy, the overall survival at 10 years was 45% in the irradiated group and 36% in the non-radiated group [22]. Our study corroborates this, showing a significant improvement in 10-year overall survival in the women taking hormonal therapy who underwent radiation. However, our study did not demonstrate the benefit of radiation to those who had targeted or chemotherapy. Also, while radiation trended towards improving overall survival in the group with only hormonal therapy without any intravenous systemic therapy, this was not statistically significant (*p* = 0.072). This could be due to the small numbers within the subgroup analysis or might show that the most important treatment in this N1-positive group of patients is intravenous systemic therapy for overall disease control (*p* = 0.010). Radiation likely provided some locoregional control on top of the effect of the hormonal therapy. A 30-year follow-up of the entire group of patients from the DBCG 82bc studies showed an overall survival of 86% in the irradiated group compared to 81% in the non-irradiated group [23]. This long-term advantage may be less relevant to older post-menopausal women whose life expectancy is often limited by other co-existing morbidities. However, the magnitude of benefit is somewhat difficult to ascertain in this group.

The PORT-N1 randomized controlled clinical trial which commenced in 2022, comparing whole breast radiation or post-mastectomy radiation for N1 disease, and whole breast radiation alone only for breast-conserving surgery patients or no radiation at all for mastectomy patients, will shed further light on the utility of post-mastectomy radiation [30]. 

It would appear that the benefit conferred by post-operative nodal radiation becomes more apparent in the long term. In the EBCTCG meta-analysis, a greater reduction in locoregional recurrence was observed at 10 years compared to at 5 years, although this benefit was not observed in our study [2]. On one hand, newer treatments confer better disease control and may reduce the absolute benefit from nodal radiation, but on the other hand, even a small benefit may be significant in these women since they are more likely to receive suboptimal systemic treatments because of advanced age and co-morbidities. This, however, remains debatable as others have found no correlation between radiation benefits and other systemic treatments [2,6]. 

Cardiac and pulmonary morbidity is significantly reduced with newer radiation techniques [31]. While there is no data to suggest that the magnitude of radiation-induced toxicity is greater in older women, older women tend to tolerate side effects less well and they are also more likely to have pre-existing ischemic heart disease and risk factors such as hypertension, diabetes, and hyperlipidemia. Dermatitis and pneumonitis are more common in women treated with post-operative nodal radiation [5], and the risk of lymphedema is also increased since full axillary nodal dissection would have been performed in most cases [32]. These side effects may not be life-threatening, but they can adversely affect the quality of life. Cardiac morbidity and secondary cancers are unlikely to be a significant problem in post-menopausal women given that these occur almost 15 to 20 years later [28], but meta-analyses did find an increase in non-cancer deaths among women with N1 disease treated with post-operative nodal radiation; therefore, radiation-related toxicity cannot be considered negligible [2]. 

Our observation that post-operative nodal radiation was associated with unfavorable risk factors, including younger age, a higher tumor grade, a greater number of involved nodes, and lymphovascular invasion, reflected the practice of selecting patients deemed to be at a higher risk of recurrence [33]. Various studies have recommended that patient age, life expectancy, tumor size, grade, lymphovascular invasion, and nodal burden be considered in the discussion for post-operative nodal radiation, and risk-predictive models incorporating these have been suggested [15,34,35,36]. Radiation oncologists at our unit seem more inclined to push for post-operative nodal radiation in women with ER-negative tumors, a practice that can be substantiated by data from the study by Whelan and colleagues which showed survival benefit to be greatest among women with ER-negative tumors who received radiation [5]. However, others have reported a greater benefit seen in women with ER-positive tumors instead [6,21]. We found no correlation with ER status in our study. In addition, the extent of nodal involvement did not appear to correlate with a 10-year outcome, although there was an initial worse outcome in the 5-year overall survival in those with three positive nodes who did not undergo irradiation. While it seemed to contradict the current practice of making a stronger recommendation in women with more than a single involved node, it may also reflect the effectiveness of systemic treatment in improving overall outcomes. In fact, reductions in recurrence and mortality were reportedly similar regardless of the number of nodes involved [2,6,22]. We did identify tumor grade and lymphovascular invasion as independent predictors of disease recurrence, but nodal radiation did not improve recurrence-free or overall survival among women with LVI-positive tumors or higher tumor grades.

This is a retrospective study. As such, there are limitations, such as selection bias in the group of women who went for radiation. There is also a risk of Type 2 error. We also recognize that our study numbers are limited, and collaboration to perform a multi-center study to study greater numbers may improve the accuracy of the study and decrease Type II errors. Furthermore, a longer follow-up will be beneficial as disease recurrence and overall survival effects in early breast cancer may only be seen later.

## 5. Conclusions

Post-menopausal women with N1 involvement and early breast cancer, with unfavorable risk factors, were perceived to be at a higher risk of recurrence and often received post-operative nodal radiation. Nodal radiation did not significantly improve 5-year or 10-year recurrence-free or overall survival. Therefore, there may not be significant benefits from radiotherapy outcomes in early breast cancer patients with N1 disease. However, in patients on hormonal therapy, radiotherapy was beneficial in improving overall survival and should be given in the adjuvant setting. Higher tumor grade and lymphovascular invasion were independent predictors of recurrence, but nodal radiation failed to significantly improve recurrence-free or overall survival in these groups of women.

## Figures and Tables

**Table 1:** Patient demographics and tumor characteristics in those who received radiotherapy (RT) compared to those who did not receive radiotherapy (RT).

	RT (N = 95)	No RT (N = 96)	*p* Value
Median Age (years)	56 (50 to 79)	65 (50–90)	<0.001
Ethnicity			0.636
Chinese	78	83	
Indian	3	5	
Malay	12	4	
Others	2	4	
Co-morbidities			0.747
Yes	68	71	
No	27	25	
Number of involved nodes			0.004
1	47	65	
2	25	21	
3	23	10	
Histology			0.331
Invasive ductal carcinoma	91	88	
Invasive lobular carcinoma	2	5	
Other histologies	2	3	
Median tumor size (mm)	23 (5–50)	25 (2–45)	0.941
Tumor grade			0.001
1	7	9	
2	24	51	
3	63	35	
Lymphovascular invasion			0.001
Present	44	22	
Absent	45	68	
ER status			0.025
Positive	66	80	
Negative	29	15	
HER2 status			0.246
Positive	20	16	
Negative	44	57	
Triple-Negative			0.127
Yes	11	6	
No	53	67	
Type of surgery			<0.001
Wide local excision	47	9	
Mastectomy	48	87	

**Table 2:** Logistic regression model stratified by disease recurrence at 10 years in women treated with post-operative nodal radiation (n = 191).

Parameter	Odds Ratio	SE	*p* Value	95% CI
Age	1.005	0.312	0.859	0.946–1.069
High tumor grade	3.038	1.363	0.013	1.261–7.317
Lymphovascular invasion	2.975	1.360	0.017	1.215–7.286
Number of involved nodes	0.307	0.252	0.150	0.062–1.530
Tumor ER status	1.887	2.361	0.612	0.163–21.912
Chemotherapy	0.392	0.216	0.089	0.134–1.152
Targeted therapy	0.803	0.573	0.758	0.198–3.245
Hormonal therapy	1.206	1.386	0.871	0.127–11.476

**Table 3:** Log-rank test and hazard ratios depicting 10-year overall survival stratified by tumor grade, lymphovascular status, nodal status, and post-operative nodal radiation (n = 191). RT: radiotherapy; LVI: lymphovascular invasion.

Comparison	*p* Value	HR	95% CI
RT vs. no RT	0.203	0.660	0.349–1.25
No RT, 3 nodes vs. 1/2 nodes	0.324	2.180	0.464–10.2
3 nodes, RT vs. no RT	0.321	0.432	0.0824–2.270
No radiation, Mastectomy, grade 3 vs. grades 1/2	0.040	2.66	1.05–6.76
Mastectomy only, Grade 3, RT vs. No RT	0.251	0.587	0.237–1.46
RT, grade 3 vs. grades 1/2	0.015	3.68	1.28–10.5
Grade 3, RT vs. no RT	0.164	0.561	0.248–1.27
No RT, LVI vs. no LVI	0.069	1.150	0.402–3.30
LVI, RT vs. no RT	0.909	1.07	0.334–3.43
Hormonal therapy, RT vs. no RT	0.047	0.443	0.199–0.988
Systemic therapy, RT vs. no RT	0.235	0.656	0.327–1.32

## Data Availability

The data presented in this study are available upon request from the corresponding author due to privacy.
